# Association between psoriasis and asthma: a systematic review and bidirectional meta-analysis

**DOI:** 10.1186/s12890-024-03078-7

**Published:** 2024-06-24

**Authors:** Doudou Wu, Xiangnan Zhou, Fan Wu, Rui Cai, Jiayi Liu, Yanping Bai

**Affiliations:** 1 Department of Integrative Traditional Chinese and Western Medicine, Beijing University of Chinese Medicine, Beijing, China; 2 Department of Dermatology, China-Japan Friendship Hospital, Beijing, China

**Keywords:** Psoriasis, Asthma, Risk factors, Meta-analysis, Immunology

## Abstract

**Background:**

The risk of asthma in patients with psoriasis has been identified in previous studies, but the bidirectional association between the two has not been fully explored.

**Methods:**

We thoroughly searched PubMed, Embase, and the Cochrane Library to find relevant observational studies published from the inception of these databases to October 2023. All the risk and bias assessments were analyzed by STATA 16.0. Where the heterogeneity was less than 50%, the fixed effect model was utilized. While where the level of heterogeneity was more than 50%, the random effect model was applied. Moreover, to identify publication bias, a visual funnel chart, and Egger’s test were applied.

**Results:**

A total of 12,396,911 participants from 16 studies, published between 2011 and 2023 were included in this meta-analysis. We found that psoriasis patients had a higher risk of developing asthma (OR = 1.48, 95%CI 1.28–1.68). Meanwhile, asthma patients also had a higher overall risk of developing psoriasis (OR = 1.33, 95%CI 1.23–1.44). In the subgroup analysis, we found that the type of study, age, and severity of the psoriasis were significant factors in the survey of asthma risk in psoriasis patients.

**Conclusions:**

In the present systematic review and meta-analysis, we found a bidirectional association between psoriasis and asthma with significantly increased risk. As a result, clinicians should make patients aware of the connection between the two, particularly adolescents or patients with moderate to severe psoriasis who need to be informed about the rising likelihood of developing asthma.

**Trial registration:**

Registration number CRD42023390111.

**Supplementary Information:**

The online version contains supplementary material available at 10.1186/s12890-024-03078-7.

## Introduction

Psoriasis is a chronic inflammatory skin disease. Its refractory and prone-to-relapse nature has caused considerable concern in the medical world for the past few decades. Erythema and silver scales on the trunk and limbs are its defining features, and its incidence ranges might be as high as 11.43% in the population [1]. Psoriasis is considered to be an immune-mediated disease related to IL-23 and IL-17 [2]. Psoriasis affects numerous organs and systems, including the cardiovascular system [3], metabolic syndrome [4], kidney disease [5], peripheral vascular disease [6], malignant tumors [7], inflammatory bowel disease [8], and so on, which make it a significant public health issue. Numerous causes intensify the above-mentioned disorders in prediagnosed patients [9]. While the introduction of biological agents opens up new possibilities for psoriasis treatment, early detection and intervention remain the most widespread methods of medical care. Therefore, early detection and prevention of psoriasis will benefit from identifying the risk factors associated with the disease’s pathogenesis.

Asthma is a chronic, non-infectious disease with airflow obstruction and respiratory symptoms. Anti-inflammatory and bronchodilator medications are frequently used to treat asthma to reduce recurrence and regulate symptoms. While around 4.3% of the global population has asthma, its occurrence has been shown to correlate with a country’s developmental level, age, gender, and other factors [10]. There are well-reported cases of comorbidities, including rhinitis [11], gastroesophageal reflux disease [12], obesity [13], obstructive sleep apnea [14], etc. The connection between skin-related allergies, asthma, and skin barrier dysfunction has received an increasing focus of attention recently [15, 16]. However, more research is still needed to confirm the link between psoriasis and asthma. While according to existing literature, asthma is a risk factor for psoriasis [17, 18], it has not been investigated whether psoriasis is a risk factor for asthma.

Psoriasis and asthma are both immune-mediated diseases with specific common inflammation-related cytokine-mediated mechanisms. IL-17 should be considered as a biomarker of this phenotype because it has been recently discovered that the differential genes of asthma with high IL-17 expression are the same as those modified in psoriasis [19]. This shows that psoriasis and asthma have comparable immunophenotypes. The type 2 IL-17 A pathway [20] has always been an important issue with asthma, particularly in severe cases. Based on the acknowledged role of the IL-17 family in the pathophysiology of psoriasis, asthma and psoriasis may be related. While numerous research has looked at the consequences of psoriasis and asthma, their findings have been unidirectional, with more recent studies looking at the deeper pathophysiological and immunological mechanisms of both conditions. As a result, based on existing research, we hypothesized a bidirectional association between psoriasis and asthma. Thus, we conducted this meta-analysis to evaluate the evidence for a bidirectional connection between psoriasis and asthma.

## Materials and methods

We conducted a systematic review and meta-analysis on observational studies (including cohort, case-control, and cross-sectional studies) to explore the bidirectional association between psoriasis and asthma. This systematic review and meta-analysis followed the guidelines of the Preferred Reporting Items for Systematic Reviews and Meta-Analyses (PRISMA) [21]. The protocol was pre-registered in the International Prospective Register of Systematic Reviews (PROSPERO) platform with the registration number CRD42023390111.

### Literature search

We searched PubMed, Embase, and the Cochrane Library for relevant studies from the databases’ inception to Oct 8th, 2023. The search strategy included medical subject headings (MeSH) and synonyms of psoriasis and asthma. There were no geographic or language restrictions. The search strategy is shown in Supplementary Table 1.

### Inclusion criteria and literature screening

The criteria for study selection were: [1] Any Cohort studies, case-control studies, or cross-sectional studies describing the prevalence or risk factors for psoriasis patients with asthma or asthma patients with psoriasis will be included [2]. If the case/exposure group is psoriasis patients, the control group comprises people without psoriasis; if the case group is asthma patients, the control group comprises people without asthma [3]. Studies of human subjects. Moreover, we excluded duplicate publications, conference abstracts, comments, letters, or studies with irrelevant results. Repeated studies on the same cohort or studies with a sample size of less than 10 were also excluded.

Study selection was performed separately by two authors (XNZ and FW) who also scanned the titles and abstracts independently and obtained the full text of potentially eligible literature. If there was a disagreement between the two authors, let the third author (YPB) make a final decision.

### Data extraction

Data extraction was performed independently by two authors (RC and JYL), and the data extraction form was designed in advance according to the guidelines of data extraction of systematic reviews and meta-analysis [22]. The baseline form of the included studies contains the first author, year, country, study type, age, follow-up years, diagnostic criteria, number of participants, adjustment, and quality assessment. In case of disagreement, the final decision was made based on a discussion or was resolved by the third author (YPB).

### Risk of bias assessment

We used the Newcastle-Ottawa scale (NOS) [23] to assess the quality of cohort studies or case-control studies. NOS scores ranged from 0 to 9, with four stars for participation versus control group selection, two for comparability, and three for outcome assessment and follow-up. Scores of 0–3, 4–6, and 7–9 indicated low, moderate, and high-quality literature, respectively.

As for cross-sectional studies, we used the recommended tools of the Agency for Healthcare Research and Quality (AHRQ) [24]. It consists of 11 items, and each item has “yes”, “no”, and “unclear” responses: “yes” equals 1 point, and “No” or “unclear” equals 0 points. The total score is 11, where 8–11 is high, 4–7 is medium, and 0–3 is low quality.

### Statistical analysis

Adjusted ORs and 95% confidence intervals (95% CI) were used to assess data, and I^2^ values were used to evaluate heterogeneity among studies within each group analysis. If *P* > 0.1 and I^2^ ≤ 50%, the fixed-effect model was used, and if I^2^ > 50% indicated large heterogeneity, the random-effect model was used. When there was too much heterogeneity, a sensitivity analysis was performed, and a recalculation was utilized after excluding the literature with obvious heterogeneity. In order to circumvent publication bias, we observed the funnel plot and used Egger’s regression to test the specific value of publication bias. If the result is > 0.05, there is no publication bias, and if it is < 0.05, it will be adjusted following the trimming method. Stata 16.0 (Stata Corp, College Station, Texas) was used for data analysis of the association between psoriasis and asthma.

## Results

### Study characteristics

**Fig. 1:**
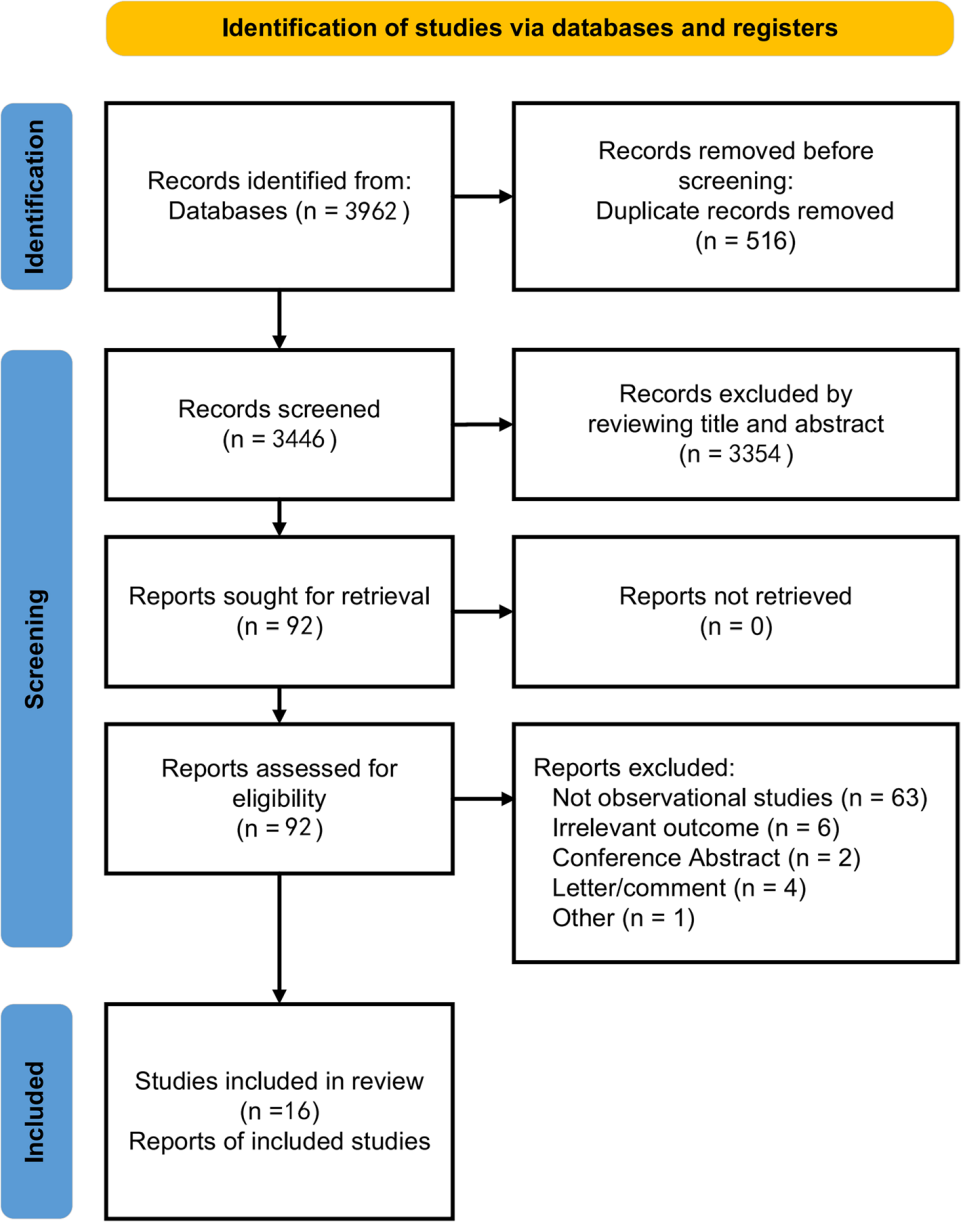
the PRISMA flow chart of study selection

Figure 1 shows the PRISMA flow chart of study selection. We retrieved 3962 records in our database search. After removing the 516 duplicates, 3446 records were left for screening. By reading the titles and abstracts, according to our inclusion and exclusion criteria, we excluded 3354 irrelevant articles. After reading the remaining 92 articles, we concluded that 16 studies [25–40] were relevant and included in the analysis.

The 16 studies were conducted between 2011 and 2023, with a total of 16,657,369 subjects included. Two cohort studies [26, 28] and ten cross-sectional studies [25, 27, 29–36] looked at the prevalence of asthma in people with psoriasis, while the prevalence of psoriasis in asthma patients was studied in four cohort studies [37–40]. The main characteristics of the included studies are summarized in Table 1.

**Table 1:** Study characteristics

Author	Year	Country	Study Type	Age(Mean ± SD)	Follow-up years	Diagnostic criteria	No. of participants	Adjustment	Quality assessment
**Studies investigating the odds of asthma in psoriasis patients**									
Martin	2022	USA	cross-sectional study	with asthma 45.3 ± 0.51/without astma 47.6 ± 0.36	NA	self-report	17,518	Sex, age, history of tobacco use, BMI, annual household income, ethnicity, and history of COPD.	6
Fang	2015	Taiwan	retrospective population-based cohort study	43.5 ± 17.0	6.37 ± 3.75	Psoriasis: ICD9-CM codes 696, 696.0, 696.1 and 696.8 Asthma: ICD-9-CM code 493	51,440	Age, sex, comorbidities	8
Yang	2011	Taiwan	cross-sectional study	30–59	NA	Psoriasis: ICD-9-CM 696.1 or 696.0	6740	(sex and age group) adjusted for monthly income, geographical region and level of urbanization of the patient’s community	8
Tsai	2011	Taiwan	retrospective cohort study	46.4 ± 18.6	6 averages	Psoriasis: ICD-9-CM 696.0–1 Asthma: ICD-9-CM 493.0、493.1、493.9	259,000	gender, age and urbanization level of the residential area	8
Galili	2017	Israel	population-based cross-sectional study	16–18	NA	NA	115,887	NA	6
Radtke	2017	Germany	cross-sectional study	> 18	NA	Psoriasis: ICD-10 L.40 Asthma: ICD-10 L.20	3217	NA	5
Hajdarbegovic	2013	Netherlands	cross-sectional study	49 ± 15.5	NA	By certified dermatologists questionnaire	280	age, sex, methotrexate use, and current smoking	8
Augustin	2015	Germany	Population-based cross-sectional study	0–18	NA	ICD-10	293,181	NA	7
Lønnberg	2015	Denmark	Population-based cross-sectional study	20–71	NA	ICD-10 L40.0-9 and self-report	33,378	NA	6
Galili	2020	Israel	Population-based cross-sectional study	16–18	NA	By relevant specialist (dermatologist, pulmonologist, otolaryngologist or allergist)	887,765	age, sex, country of origin, socioeconomic status, number of siblings and body mass index	9
Tanimura	2023	USA	cross-sectional study	40–69	NA	Psoriasis: ICD-10 M07 and L40 Asthma: ICD-10 J45	472,782	age, sex, weight, diabetes mellitus, and smoking history	7
Joel	2023	USA	cross-sectional study	54.7 ± 16.6	NA	SNOMED Psoriasis: 9,014,002 Asthma: 195,967,001	235,551	age, sex, race/ethnicity, body mass index, annual household income, and smoking status	7
**Studies investigating the odds of psoriasis in asthma patients**									
Han	2021	Korea	cohort study	> 20	8 averages	Asthma: J45–46 Psoriasis: ICD-10 L40	9,718,722	Age, sex, smoking, alcohol consumption, physical activity, income level and body mass index	8
Kim	2019	Korea	cohort study	0–85+	7.15 ± 3.575	Asthma: ICD-10 J45–46 Psoriasis: ICD-10 B02	301,450	Age, sex, income, region of residence, hypertension, diabetes, and dyslipidemia	8
Egeberg	2015	Denmark	cohort study	6–14	14 averages	Asthma: ICD-10 J45 Psoriasis: ICD-10 L40	1,478,110	Age, sex, comorbidities and medications/age, sex	7
Krishna	2019	U.K	retrospective cohort	35.61 ± 21.26	28 averages	NA	2,782,348	NA	7

### Quality assessment

The NOS scale and AHRQ were used in this investigation to rate the quality of cohort and cross-sectional studies, respectively. As for the included studies of psoriasis patients with asthma, the NOS scale was used to rate the quality of the cohort studies, which both received scores of 8, and the AHRQ was used to rate the quality of the cross-sectional studies, which received scores ranging from 5 to 9, with an average of 6.9. For the studies about asthma patients with psoriasis, the NOS scale was used to evaluate the quality of four cohort studies, with scores ranging from 7 to 8, with an average score of 7.5. In summary, all studies in this meta-analysis are qualified. The specific scores are shown in Table 1.

### Bidirectional association between psoriasis and asthma

**Fig. 2:**
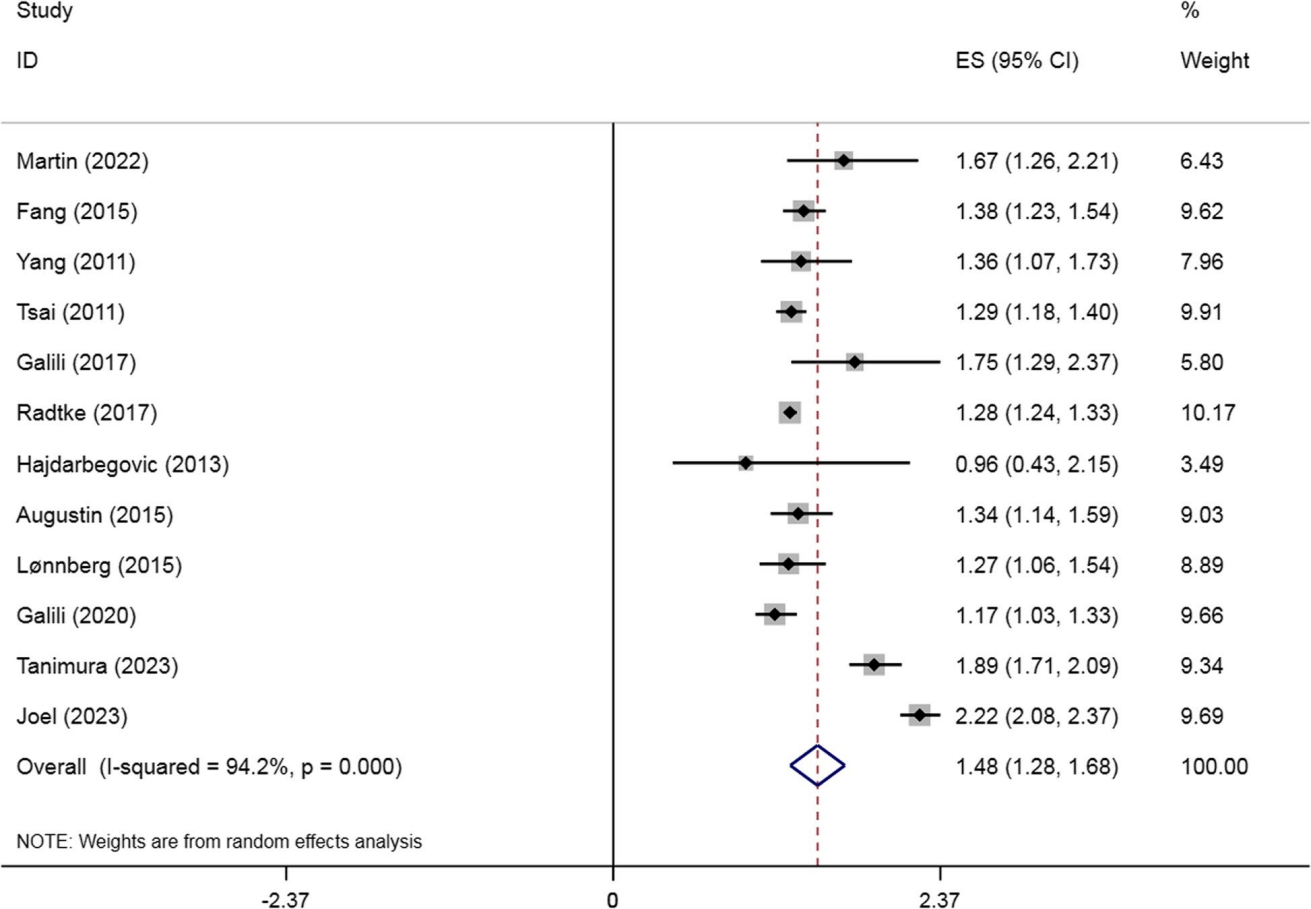
Forest plot for studies of the association on psoriasis with asthma

Two cohort studies [26–28] and ten cross-sectional studies [25, 27, 29–36] explored the risk of asthma in patients with psoriasis. According to the meta-analysis, asthma was associated with an increased incidence of psoriasis (OR = 1.480, 95% CI: 1.282–1.678, I^2^ = 95.8%, *P* < 0.001, Fig. 2). As the I^2^ of the study is over 50%, which is high heterogeneity, it is reasonable to use the random effect model.

**Fig. 3:**
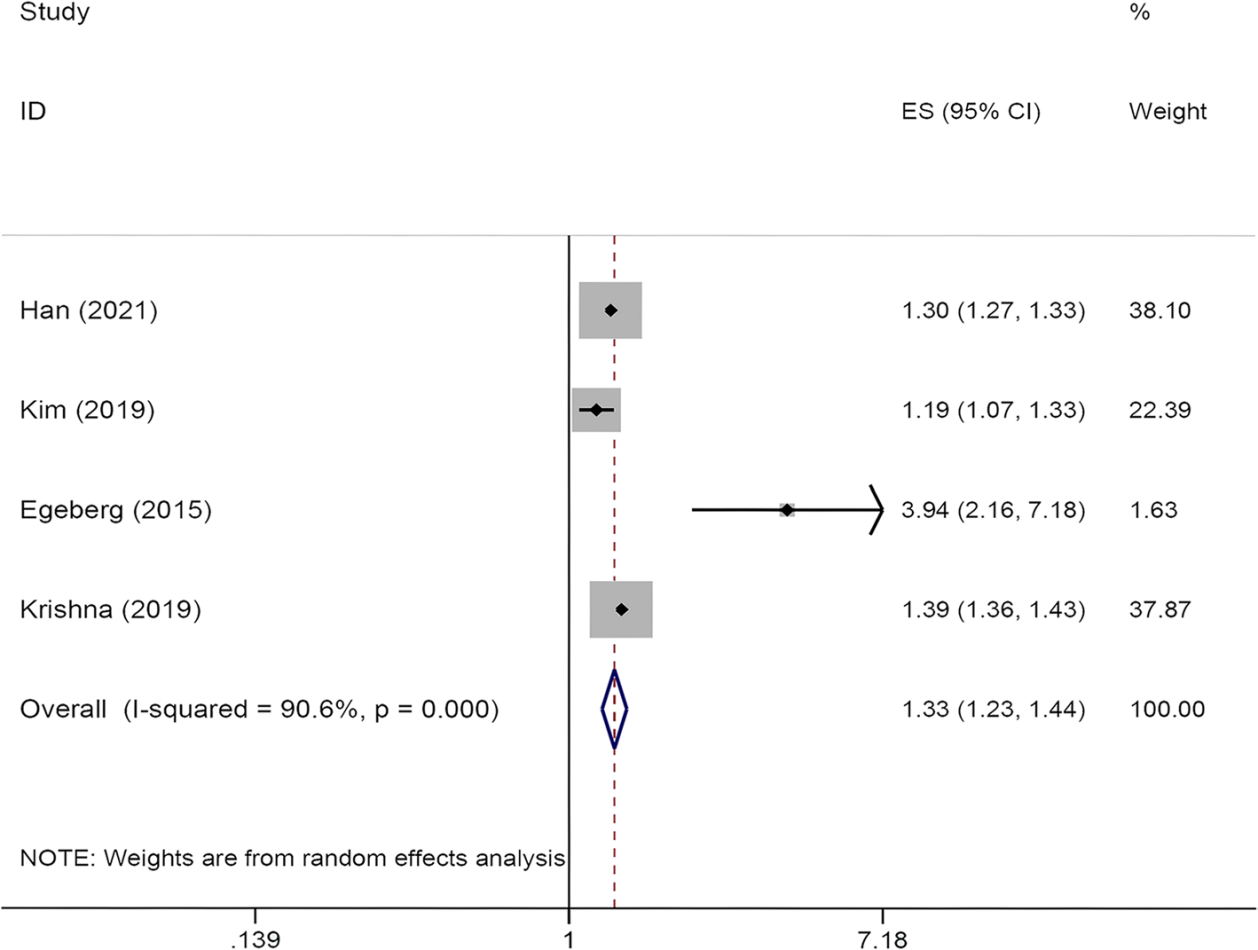
Forest plot for studies of the association on asthma with psoriasis

Four cohort studies [37–40] explored the risk of psoriasis in asthmatic patients. According to the meta-analysis, psoriasis was associated with an increased incidence of asthma (OR = 1.331, 95% CI: 1.231–1.440, I^2^ = 90.6%, *P* < 0.01, Fig. 3). However, as the I^2^ of the study is higher than 50%, to adjust for heterogeneity, the random effect model is used for analysis. As the sensitivity analysis shows, one article may lead to high heterogeneity so it was excluded from the final results. The adjusted data showed similar results.

### Subgroup analysis

We grouped 12 studies according to their type and age, and grouped 10 studies on the severity of psoriasis, focusing on the risk of asthma in patients with psoriasis. However, the risk of psoriasis among patients with asthma could not be analyzed by subgroup because of the small number of included articles. We found that the type of the study, age and severity of psoriasis is affected by asthma. The specific results are shown in Table 2. The risk of asthma in patients with psoriasis was higher in cross-sectional studies (OR = 1.489, 95% CI (1.224, 1.811)) than in cohort studies (OR = 1.322, 95% CI (1.235, 1.415)), the risk of asthma was higher in adults (OR = 1.490 95% CI (1.234, 1.799)) than in adolescents (OR = 1.344, 95% CI (1.112, 1.624)), and the risk of asthma was higher in patients with moderate to severe psoriasis (OR = 1.390, 95% CI (1.243, 1.554)) than in patients with mild psoriasis (OR = 1.235, 95% CI (1.072, 1.424)). This shows that there are significant differences between the study type, age, and psoriasis severity groups (*p* = 0.000).

**Table 2:** Subgroup analysis

Subgroups	Included Studies	OR (95%CI)	Heterogeneity	Significance test	
			I^2^(%)	Z	*P*-values
**Study Type**					
Cohort study	2	1.322 (1.235, 1.415)	0.0	3.98	0.000
Cross-sectional study	10	1.489 (1.224, 1.811)	96.5	8.05	0.000
Subgroups differences	12		95.8	4.79	0.000
**Age**					
≤ 18	3	1.344 (1.112, 1.624)	68.0	3.06	0.002
> 18	9	1.490 (1.234, 1.799)	96.8	4.15	0.000
Subgroups differences	12		95.8	4.79	0.000
**Severity of Psoriasis**					
mild	4	1.235 (1.072, 1.424)	73.3	2.91	0.004
Moderate to severe	6	1.390 (1.243, 1.554)	0.0	5.77	0.000
Subgroups differences	10		52.1	5.50	0.000

### Publication bias

**Fig. 4:**
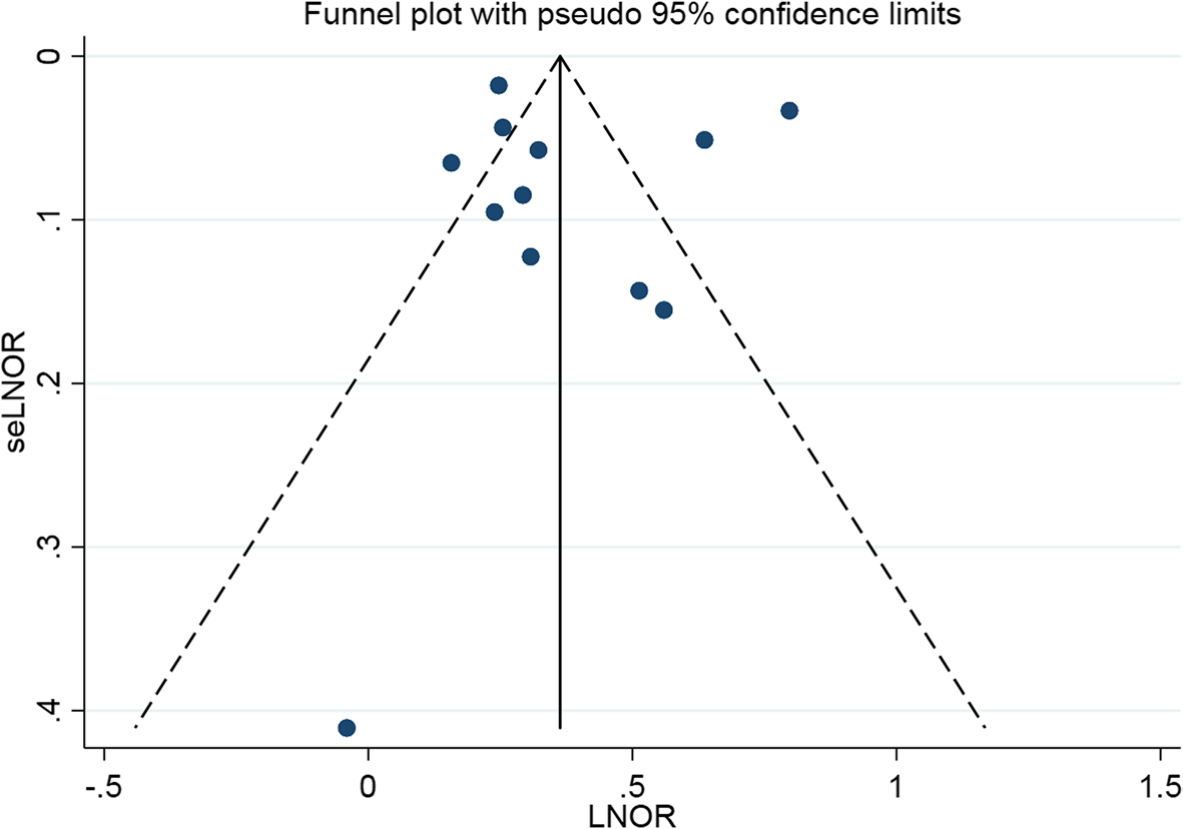
Publication bias of psoriasis patients with asthma

**Fig. 5:**
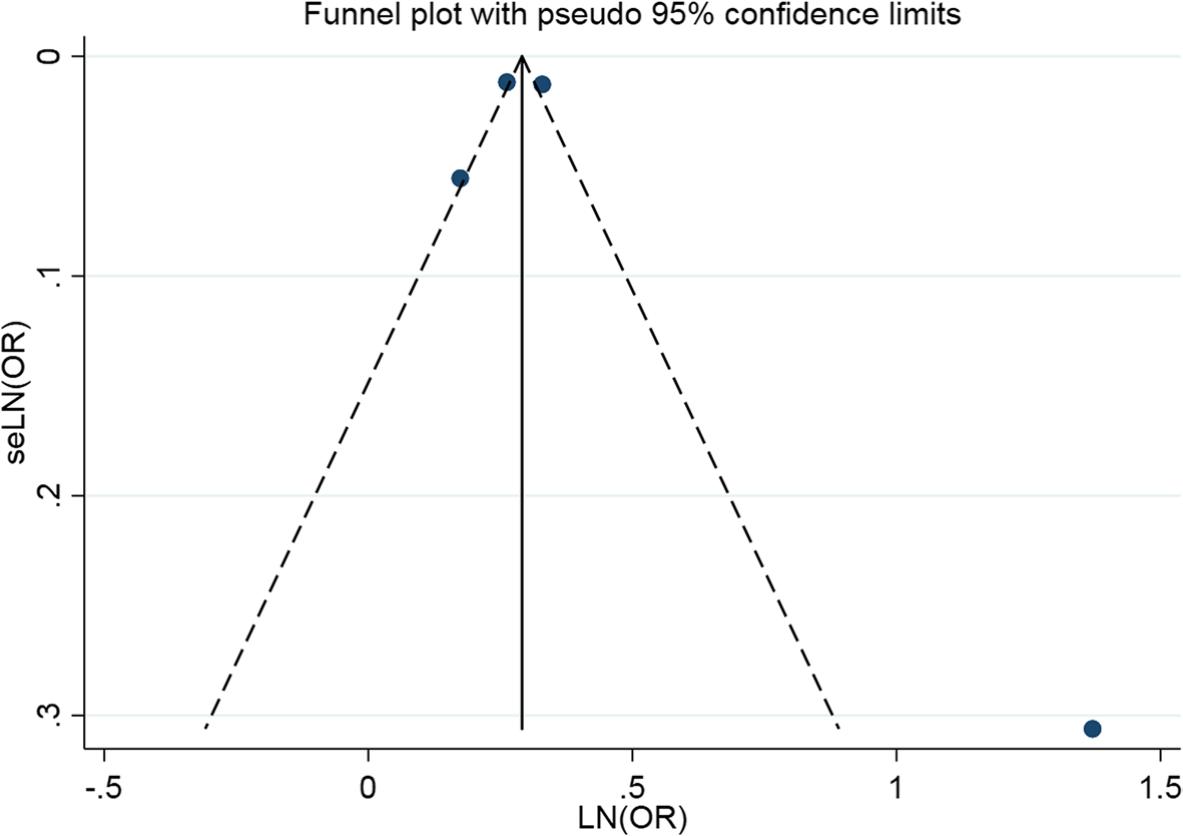
Publication bias of asthma patients with psoriasis

We tested the publication bias against funnel chart visual examination and Egger’s regression test and concluded that there was no significant publication bias in the literature included in the two-way association between psoriasis and asthma (Figs. 4 and 5). The visual results of the funnel chart and the results of two Egger’s regression tests (*P* = 0.708 and *P* = 0.716) proves that there is no evidence of publication bias.

## Discussion

### Main findings

Our meta-analysis investigated the bidirectional association between psoriasis and asthma. This meta-analysis was conducted on 16 studies, including 6 cohort and 10 cross-sectional studies, with a total subject number of 16,657,369. The final results state that asthma increases the risk of psoriasis by 1.48 times, whereas psoriasis increases the risk of asthma by 1.33 times. Concerning the effect of asthma on psoriasis patients, we conducted a subgroup analysis and found that the different ages, types of research, and severity of psoriasis were affected.

### Interpretation of the main results

Asthma increases the chance of developing psoriasis, according to the findings of two previous systematic reviews and meta-analyses [17, 18]. However, after performing a literature search, we discovered that psoriasis could also act as a risk factor for the development of asthma. As a result, there is an unstudied bidirectional relationship between psoriasis and asthma. To further explore the potential bidirectional relationship between the two, we added additional research to the previous studies that focus on the risk of psoriasis in asthmatic patients. In this systematic review and meta-analysis, we discovered a bidirectional association between asthma and psoriasis acting as mutual risk factors.

The co-action mechanism of psoriasis and asthma has attracted scholars’ attention in recent years. Traditional T helper cells are subdivided into Th1 and Th2 subgroups due to several cytokines being secreted. Psoriasis is a chronic inflammatory immune-mediated dermatosis that involves both Th1 and Th17 cells, with Th1 cells secreting IFN-γ and TNF-α and Th17 cells secreting IL-17, IL-22 and TNF-α [41]. Asthma is thought to be a chronic allergic disease that is mediated by either the Th2 subgroup or a non-Th2 subgroup (Th1 or Th17) [42]. However, due to the high degree of heterogeneity among asthmatic patients, the relationship between Th2 and non-Th2 has not been thoroughly elucidated. Among them, Th17 is an important factor related to asthma airway reaction and neutrophil infiltration [43], but its role in asthma is still unclear. In-depth phenotypic studies on asthma patients with high IL-17 expression by certain researchers revealed that this aspect of the phenotype is also present in psoriasis [19], suggesting that IL-17 may act as a link between psoriasis and asthma.

The subgroup analysis further revealed that asthma affected patients with psoriasis differently based on the different ages, severity levels, and study types. Wang [18] conducted a subgroup analysis of age and study type among psoriasis patients, and concluded that older adults were more sensitive to asthma than younger adults. According to Fang et al. [17], there was no difference between the pediatric and adult groups when it came to the increased risk of developing asthma. Interestingly, our study found that asthma susceptibility was higher in adolescents (≤ 18) (OR = 1.344, 95% CI (1.112,1.624)) than in adults (OR = 1.293, 95% CI (1.254,1.332)). However, there were only 3 publications in the adolescent group with moderate heterogeneity (68%), which may impact the finding’s accuracy. At the same time, we found that the severity of psoriasis was associated with the susceptibility to asthma to some extent. Among them, severe psoriasis (OR = 1.390, 95% CI (1.243–1.554)) is more susceptible than mild and moderate cases (OR = 1.235, 95% CI (1.072) 1.424). Therefore, the prevalence of asthma in psoriasis should be given more consideration, especially in cases of severe psoriasis.

### Implications and limitations

This study is the first to conclusively claim a bidirectional link between psoriasis and asthma, with the systematic review and meta-analysis summarizing all previous research on the topic. Consequently, this paper proposes that while making a clinical diagnosis, it is essential to consider how psoriasis and asthma differ and interact. Due to the high degree of heterogeneity and the scarcity of studies on psoriasis in asthmatic patients, there are still some limitations to the scope of this study. After performing a sensitivity analysis, the source of the heterogeneity was not identified, and subgroup analysis was not done. With additional literature, the heterogeneity could be decreased in the future. There may be certain issues, such as recall bias since this study is merely a cross-sectional and cohort study focusing on the risk of asthma in psoriasis patients. More diverse types of literature can be utilized to improve the reliability of the findings in subsequent studies.

## Conclusions

In conclusion, the evidence in support of a bidirectional relationship between psoriasis and asthma is substantial. The risk of psoriasis in asthma and the risk of asthma in psoriasis are both significant, as shown in this systematic review and meta-analysis. Consequently, patients with asthma should pay attention to the timely identification of psoriatic lesions because, according to the research available at the moment, there are similarities between the two pathogenic mechanisms. Nevertheless, more evidence is still required to support the recommendation that psoriasis patients with respiratory symptoms be tested for asthma.

### Supplementary Information

Supplementary Material 1.

## Data Availability

Data is provided within the manuscript.
