# Supplementary material for: Association between psoriasis and asthma: a systematic review and bidirectional meta-analysis
Source: BMC Pulm Med. 2024 Jun 24;24:293. doi: 10.1186/s12890-024-03078-7 (PMC11197190; doi:10.1186/s12890-024-03078-7)
Supplement: Supplementary file 1 — Supplementary Material 1. [file 12890_2024_3078_MOESM1_ESM.docx]

**Supplementary Table 1 Search strategy**

**PubMed**

| No. | Content | Result |
| --- | --- | --- |
| #1 | "Asthma"[Mesh] | 140139 |
| #2 | "Asthma*"[Title/Abstract] | 177946 |
| #3 | #1 OR #2 | 197871 |
| #4 | "Psoriasis"[Mesh] | 46797 |
| #5 | ((((("Psorias*"[Title/Abstract]) ) OR ("Pustulosis of Palms and Soles"[Title/Abstract])) OR ("Pustulosis Palmaris et Plantaris"[Title/Abstract])) OR ("Palmoplantaris Pustulosis"[Title/Abstract])) OR ("Pustular Psoriasis of Palms and Soles"[Title/Abstract]) | 61182 |
| #6 | #4 OR #5 | 43,645 |
| #7 | #3 AND #6 | 592 |

**Cochrane Library**

| No. | Content | Result |
| --- | --- | --- |
| #1 | MeSH descriptor: [Asthma] explode all trees | 12316 |
| #2 | ("Asthma*"):ti,ab,kw | 34278 |
| #3 | #1 OR #2 | 34285 |
| #4 | MeSH descriptor: [Psoriasis] explode all trees | 3739 |
| #5 | (Psorias*):ab,ti,kw OR (Pustulosis of Palms and Soles):ab,ti,kw OR (Pustulosis Palmaris et Plantaris):ab,ti,kw OR (Palmoplantaris Pustulosis):ab,ti,kw OR (Pustular Psoriasis of Palms and Soles):ab,ti,kw | 9236 |
| #6 | #4 OR #5 | 9572 |
| #7 | #3 AND #6 | 41 |

**Embase**

| No. | Content | Result |
| --- | --- | --- |
| #1 | 'asthma'/exp | 305771 |
| #2 | 'asthma*':ab,ti | 262630 |
| #3 | #1 OR #2 | 342273 |
| #4 | 'psoriasis'/exp | 110817 |
| #5 | 'psorias*':ab,ti OR 'pustulosis of palms and soles':ab,ti OR 'pustulosis palmaris et plantaris':ab,ti OR 'palmoplantaris pustulosis':ab,ti OR 'pustular psoriasis of palms and soles':ab,ti | 76085 |
| #6 | #4 OR #5 | 118056 |
| #7 | #3 AND #6 | 2839 |
